# Water-dispersible TiO_2_ nanoparticles via a biphasic solvothermal reaction method

**DOI:** 10.1186/1556-276X-8-503

**Published:** 2013-12-01

**Authors:** Rajneesh Mohan, Jana Drbohlavova, Jaromir Hubalek

**Affiliations:** 1 Central European Institute of Technology (CEITEC-BUT), Brno University of Technology, Technicka 3058/10, Brno 61600, Czech Republic

**Keywords:** TiO_2_, X-ray diffraction, UV-vis absorption, Fluorescence spectra

## Abstract

A biphasic solvothermal reaction method has been used for the synthesis of TiO_2_ nanoparticles (NPs). In this method, hydrolysis and nucleation occur at the interface of organic phase (titanium (IV) *n*-propoxide and stearic acid dissolved in toluene) and water phase (*tert*-butylamine dissolved in water) resulting in the nucleation of the stearic acid-capped TiO_2_ NPs. These NPs are hydrophilic due to hydrophobic stearic acid ligands and could be dispersed in toluene, but not in water. These stearic acid-capped TiO_2_ NPs were surface-modified with 2,3-dimercaptosuccinic acid (DMSA) in order to make them water soluble. The resultant TiO_2_ NPs were easily redispersed in water without any noticeable aggregation. The Rietveld profile fitting of X-ray diffraction (XRD) pattern of the TiO_2_ NPs revealed highly crystalline anatase structure. The average crystallite size of TiO_2_ NPs was calculated to be 6.89 nm, which agrees with TEM results. These results have important implications for the use of TiO_2_ in biomedical, environmental, and industrial applications.

## Background

TiO_2_ nanoparticles (NPs) have been widely investigated in the recent past due to their applications in a wide range of fields including solar cells [1], water photolysis for hydrogen production [2], sensors [3], and antireflective and photochromic devices [4]. TiO_2_ has three well-known crystallographic phases in nature: anatase, rutile, and brookite. Among these, anatase has been proved to have excellent chemical and physical properties for environmental remediation [5] and many other uses [6-8]. Numerous methods for the synthesis of TiO_2_ NPs have been developed, such as hydrolytic sol-gel process [9], nonhydrolytic sol-gel process [10], hydrothermal methods [11], solvothermal methods [12], and so on. The synthesis of TiO_2_ nanoparticles generally involves hydrolysis and condensation of titanium precursors. The titanium precursors are extremely water sensitive; therefore, in conventional aqueous/alcohol-phase/sol-gel method in conventional solution-phase synthetic routes, small amount of water is used to inhibit the hydrolysis. However, prepared TiO_2_ NPs suffer from poor crystallinity and inferior material properties as compared to those prepared through high-temperature, nonhydrolytic methods. Furthermore, these methods of synthesis suffer from problems of aggregation, size, and low monodispersity and post-treatment procedures (for converting amorphous phase to crystalline TiO_2_ phase) which greatly affect the desired properties of the nanoparticles and restrict their large-scale production and applicability. The properties of TiO_2_ are highly dependent on surface area, crystalline phase, and single crystallinity. The high-quality TiO_2_ NPs prepared through nonhydrolytic methods are insoluble in aqueous medium, which make their utilization toward biological/biomedical applications impossible. At present, the synthesis methods for production of water-dispersible TiO_2_ NPs with a tunable size is challenging to the researchers.

In this letter, we present the preparation of water-soluble and biocompatible highly crystalline TiO_2_ NPs through biphasic solvothermal interface reaction method.

## Methods

The following chemicals were used as purchased: titanium (IV) *n*-propoxide, *tert*-butylamine, 2,3-dimercaptosuccinic acid (DMSA) and stearic acid (SA) (Sigma-Aldrich, Steinheim, Germany) and toluene (Penta, Chrudim, Czech Republic). All the chemicals were of analytical grade purity. Deionized water (Millipore) was used to prepare aqueous solutions (≥18 MΩ). In biphasic solvothermal reaction method, the reaction occurs at the interface of water phase and organic phase at elevated temperature. In the synthesis procedure, the organic phase consists of 90 μL of titanium (IV) *n*-propoxide and 0.5 g of SA dissolved in 10 mL of toluene. The water phase contains 100 μL of *tert*-butylamine dissolved in 10 mL of deionized (DI) water. First, water phase was added to a Teflon-lined steel autoclave. Then, the organic phase was added slowly into the Teflon-lined steel autoclave without any stirring. The autoclave was sealed and heated to 170°C for 6 h. The reaction mixture was then cooled to room temperature, and methanol was added to precipitate the TiO_2_ NPs. TiO_2_ NP precipitates were recovered by centrifugation and washed several times with methanol to remove the excess of surfactant. This resulted in hydrophobic SA-coated TiO_2_ NPs, which are dispersible in toluene. The water dispersiblity of TiO_2_ NPs was achieved by treating the SA-coated TiO_2_ NPs in a solution of ethanol and toluene containing 2,3-DMSA for 24 h with vigorous stirring. This resulted in DMSA-coated TiO_2_ NPs which were recovered via centrifugation. Then, the final NPs were easily dispersed in water.

The crystal structure and morphology of as-synthesized nanoparticles were investigated with X-ray diffraction (XRD) using monochromatic Cu Kα radiation (*λ* = 1.5418 Å) and transmission electron microscope (TEM). The crystalline nature of the NPs was then examined by TEM measurements. The optical properties were investigated by UV-visible (UV-vis) absorption and fluorescence spectra at room temperature.

## Results and discussion

**Figure 1:**
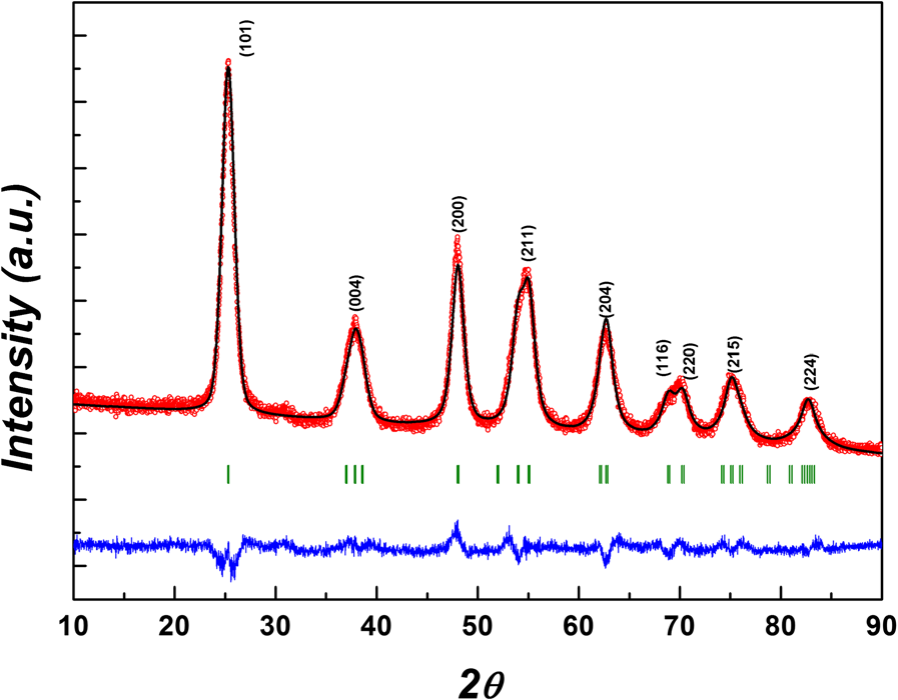
**The Rietveld profile fitting of X-ray diffraction pattern of pure anatase TiO**_**2 **_**NPs.** Experimental (symbols) and fitting (solid lines) X-ray diffraction patterns. The positions of Bragg reflections are denoted by vertical bars. The difference (experiment minus calculation) curve is shown by a solid line at the bottom.

During heating, hydrolysis and nucleation of the titanium (IV) *n*-propoxide occur at the interface of organic phase and water phase resulting in simultaneous nucleation of TiO_2_ NPs. The XRD pattern of TiO_2_ NP sample prepared at 170°C was analyzed with Rietveld profile fitting method using FullProf program [13] within anatase I41/amd space group. The Rietveld profile fitting of XRD pattern of prepared TiO_2_ NPs illustrated in Figure 1 shows the good quality of the fit. No impurity phases were found in the XRD patterns of TiO_2_ NP samples. The diffraction peaks were indexed with powder diffraction standard data (ICDD 21-1272). The crystallite size of TiO_2_ NPs is estimated from broadening of anatase (101) peak using the Debye-Scherrer formula [14]. The calculated crystallite size for TiO_2_ nanoparticles prepared at 170°C is 6.89 nm. The nanoparticles were also prepared at lower temperatures (140°C, 150°C, and 160°C) and higher temperatures (180°C and 190°C). NPs prepared at lower temperatures have smaller crystallite size but the product yield is low, while NPs prepared at higher temperatures have higher yield but the crystallite size is bigger. The optimum temperature is 170°C for the preparation of TiO_2_ NPs with narrow size distribution and nearly 100% yield.

**Figure 2:**
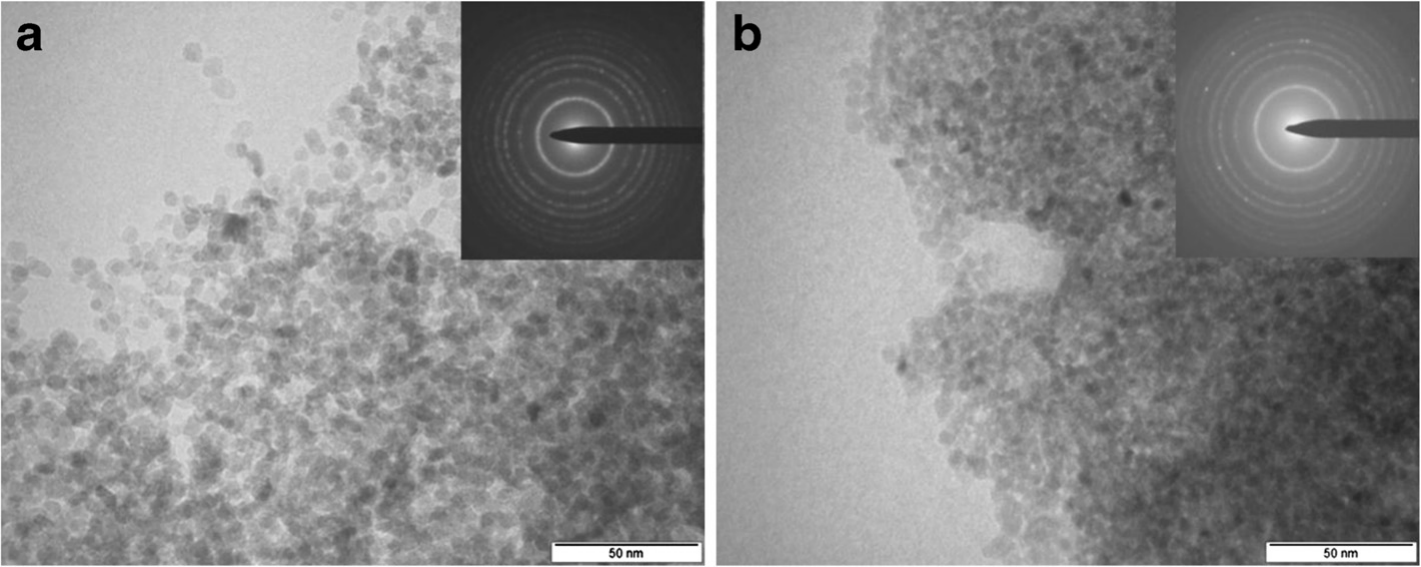
**TEM image of the TiO**_**2 **_**NPs. (a)** Toluene-dispersible SA-coated NPs. **(b)** Water-dispersible DMSA-coated NPs. The insets show the corresponding electron diffraction patterns.

The morphology of SA-coated and DMSA-coated TiO_2_ NPs was examined by TEM measurements. As shown in Figure 2a,b, the resulting TiO_2_ NPs (SA-coated and DMSA-coated) appear as spherical particles with good monodispersity. The size distribution of the nanoparticles is in Additional file 1: Figure S2, calculated by measuring hundred particles, shows that the TiO_2_ NPs have an average size of 6 nm, which is in good accordance with the size of TiO_2_ NPs observed through XRD measurement. The inset of Figure 2a,b presents the SAED pattern of TiO_2_ NPs, confirming that anatase crystal structure can be indexed with (101), (103), (200), (105), (213), (116), (107), and (008) crystallographic planes.

**Figure 3:**
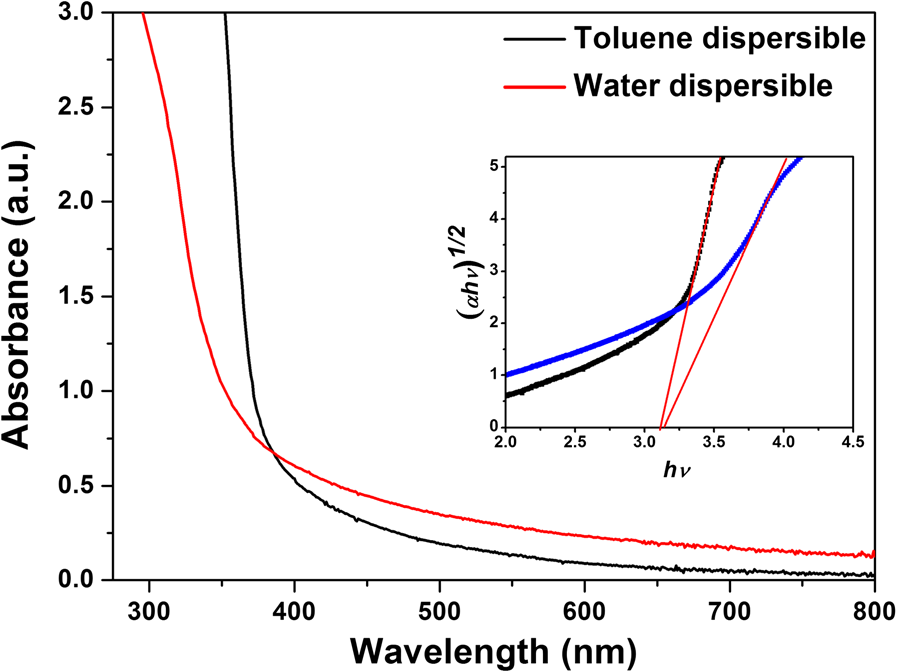
**UV-visible absorption spectra of TiO**_**2 **_**NP dispersion in toluene and DI water.** The inset shows the corresponding plots of (*αhν*)^1/2^ as a function of photon energy.

UV-vis absorption spectra of TiO_2_ nanoparticles dispersed in toluene and DI water are measured and shown in Figure 3. The absorption coefficient (*α*) was determined from the optical spectrum using the formula

$$\alpha =2.3026\frac{A}{t},$$

where *A* and *t* are the measured absorbance and thickness of the sample, respectively. The optical bandgap energy (*E*_g_) was evaluated from the absorption spectrum, and the optical absorption coefficient (*α*) near the absorption edge is given by following equation:

$$\mathrm{\alpha h\nu}=B{\left(\mathrm{h\nu}-E_{g}\right)}^{n},$$

where *h*, *ν*, *B*, and *E*_g_ are Plank's constant, frequency of incident photons, constant, and optical bandgap energy, respectively. *E*_g_ was estimated by plotting *hν* versus (*αhν*)^1/2^ and extrapolating linear portion near the onset of absorption edge to the energy axis as shown in the inset of Figure 3. The determined value of *E*_g_ is 3.06 and 3.1 eV for TiO_2_ nanoparticles dispersed in toluene and DI water, respectively.

**Figure 4:**
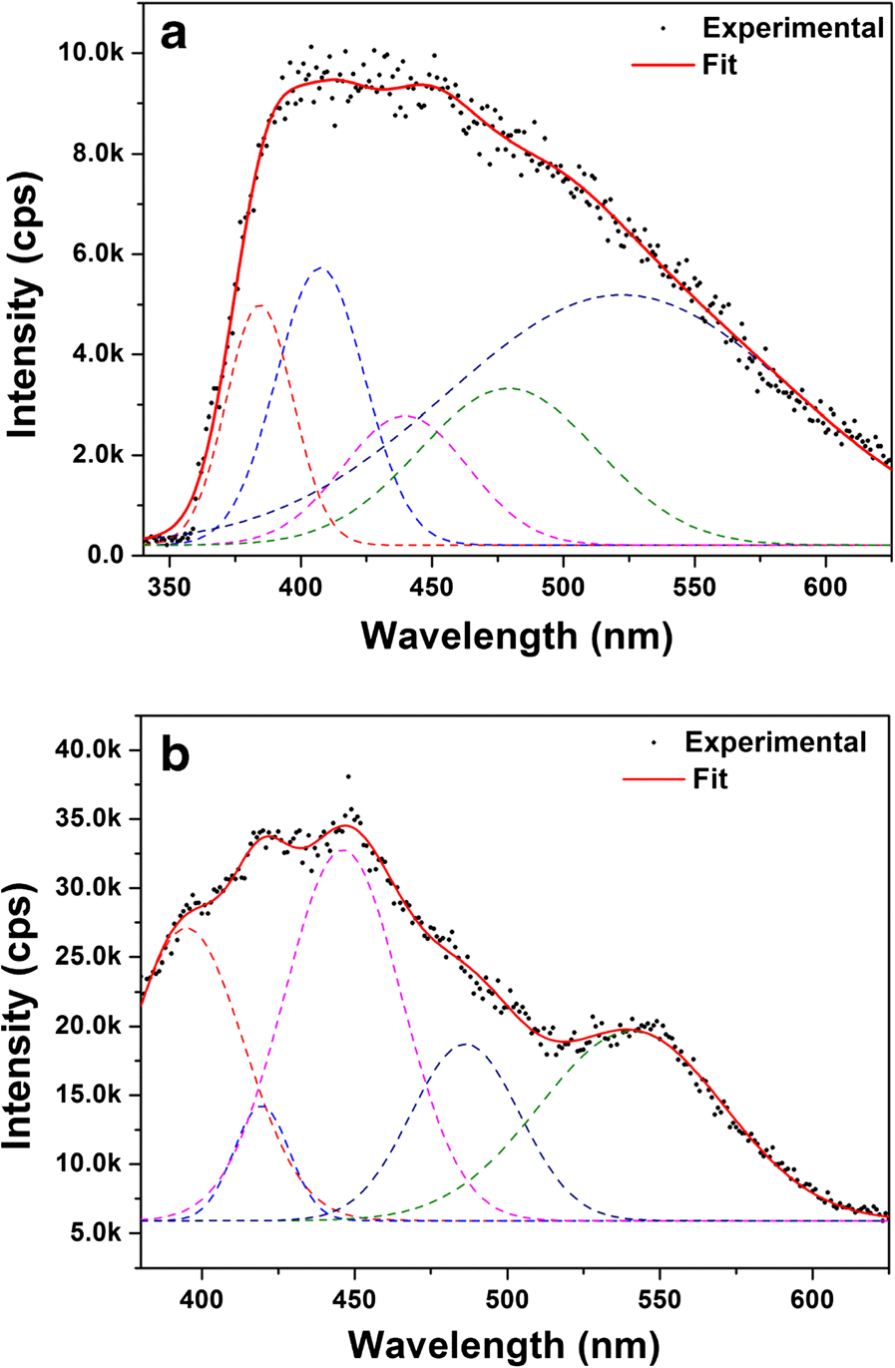
**Fluorescence spectra of TiO**_**2 **_**NP. (a)** Toluene-dispersible SA-coated NPs. **(b)** Water-dispersible DMSA-coated NPs. The fluorescence spectra are deconvoluted into Gaussian line shapes. The experimental data are shown in solid circles. The dashed lines correspond to the individual components by Gaussian fitting, and the solid lines represent the sum of individual fitting lines.

Fluorescence spectra of SA-coated TiO_2_ NPs in toluene and DMSA-coated TiO_2_ NPs in DI water with an excitation wavelength of 325 nm were recorded at room temperature and are shown in Figure 3a,b. The broad emission spectra which are observed from 400 to 500 nm arise from indirect bandgap and surface recombination processes [15]. After multipeak Gaussian fitting of fluorescence spectra in Figure 3a,b, we found that Gaussian curves fit original curves perfectly. The peak positions of Gaussian bands in Figure 4a are located at about 384, 407, 440, 480, and 525 nm, respectively. The peak positions of Gaussian bands in Figure 4b are located at about 394, 418, 445, 485, and 540 nm, respectively. All these peaks are red shifted due to the light-induced relaxation of polar molecules [16]. The prepared TiO_2_ NPs with high surface-to-volume ratio favor the existence of large quantities of oxygen vacancies. The observed fluorescence bands may be the result of emission from radiative recombination of self-trapped excitons localized within TiO_6_ octahedra and oxygen vacancies [17]. Oxygen vacancies have been considered as the most common defects and usually act as radiative centers in the luminescence processes [18]. The emission peak at about 384/394 nm is attributed to the emission of near bandgap transition of anatase. This is consistent with the *E*_g_ calculated by UV measurement techniques (i.e., approximately 3.1 eV). The emission bands at 407 and 418 nm were ascribed to electron transition mediated by defect levels in the bandgap [19]. In addition, the signals observed in wavelength range from 440 to 540 nm arise from the excitonic PL, which mainly results from surface oxygen vacancies and defects. The peaks at 440 and 445 nm are attributed to band edge free excitons, and the other peaks at 480 and 485 nm corresponds to bound excitons [20].

## Conclusions

A facile route for the synthesis of TiO_2_ NPs through biphasic solvothermal interface reaction method has been reported. The XRD pattern of TiO_2_ NPs revealed the anatase structure. The average XRD crystallite size was calculated as 6.89 nm using the Scherrer formula. The optical studies showed that the bandgap is 3.1 eV. The results show that synthesized nanoparticles are monodispersed with long-term stability. This synthesis method is simple with very high production yield and does not require any post-treatment procedure, and products can be collected from organic phase which effectively avoids TiO_2_ grain aggregation.

## Competing interests

The authors declare that they have no competing interests.

## Authors’ contributions

RM carried out the synthesis and characterization. JD improved the manuscript and participated in the studies. JH directed and coordinated the present study as principal investigator. All authors read and approved the final manuscript.

## Supplementary Material

Additional file 1 **Synthesis, size distribution, XRD patterns, and FTIR spectra of TiO**_**2 **_**nanoparticles. Figure S1:** Schematic of TiO_2_ nanoparticles synthesis via a biphasic solvothermal interface reaction method. **Figure S2:** The size distribution of the nanoparticles. **Figure S3:** The XRD patterns of the TiO_2_ nanoparticles prepared at different temperatures. **Figure S4:** FTIR spectra of the SA-capped TiO_2_ nanoparticles. Click here for file
