# Supplementary material for: Water-dispersible TiO2 nanoparticles via a biphasic solvothermal reaction method
Source: Nanoscale Res Lett. 2013 Dec 1;8(1):503. doi: 10.1186/1556-276X-8-503 (PMC4219175; doi:10.1186/1556-276X-8-503)
Supplement: Additional file 1 — Synthesis, size distribution, XRD patterns, and FTIR spectra of TiO2 nanoparticles. Figure S1: Schematic of TiO2 nanoparticles synthesis via a biphasic solvothermal interface reaction method. Figure S2: The size distribution of the nanoparticles. Figure S3: The XRD patterns of the TiO2 nanoparticles prepared at different temperatures. Figure S4: FTIR spectra of the SA-capped TiO2 nanoparticles. [file 1556-276X-8-503-S1.docx]

**Additional File**

**Water dispersible TiO2 nanoparticles via a biphasic solvothermal reaction method**

**Rajneesh Mohan, Jana Drbohlavova and Jaromir Hubalek**

Central European Institute of Technology, Brno University of Technology, Technicka 10, 61600 Brno, Czech Republic

**Schematic of the synthesis of TiO_2_ nanoparticles via a biphasic solvothermal interface reaction method**

When the organic phase consisting of titanium (IV) n-propoxide and stearic acid (SA) in toluene added slowly to the water phase containing tert-butylamine in 10 ml of deionized (DI) water, an interface is created due to deferent densities as shown in Fig. S1. This interface does not allow the uncontrolled instantaneous hydrolysis of titanium precursor. When heated in a closed vessel, the reaction starts at the interface which spread throughout the reaction vessel at elevated temperature. Because of the lower boiling point of water, the water vapor filled the whole reaction vessel. This resulted in controlled hydrolysis of titanium precursor.


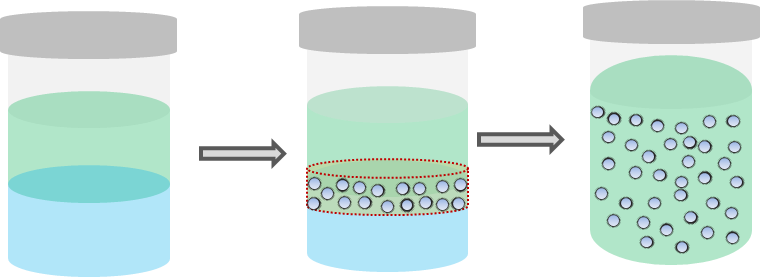


**Fig S1:** Schematic of the synthesis of TiO_2_ nanoparticles via a biphasic solvothermal interface reaction method


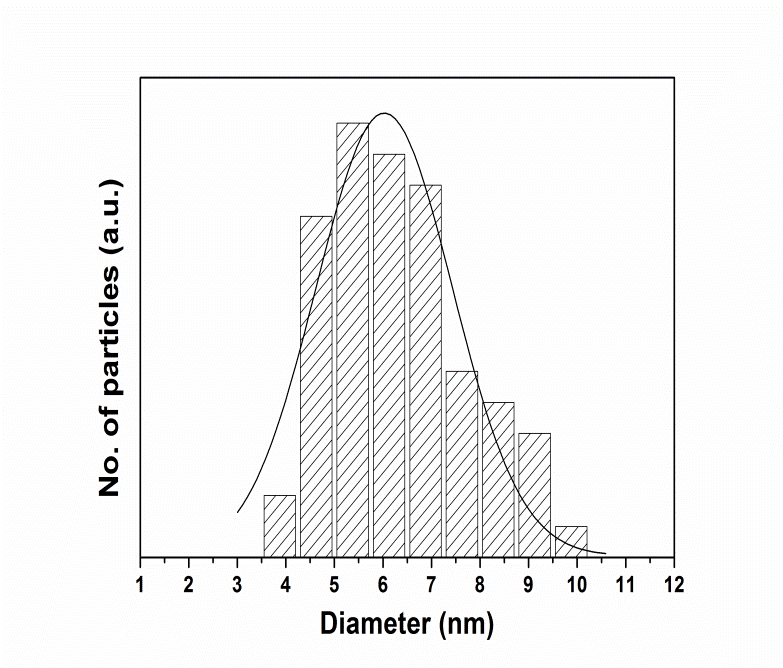


**Fig S2: The size distribution of the TiO_2_ nanoparticles.**

**The size distribution of the nanoparticles**

The size distribution of the nanoparticles in Fig. 2S, calculated by measuring hundred particles, shows that the TiO_2_ NPs have an average size of 6 nm, which is in good accordance with the size of TiO_2_ NPs observed was XRD measurement**.**

**XRD Analysis:**

The XRD patterns of the TiO_2_ nanoparticles prepared at different temperatures are illustrated in Fig. S3. The diffraction peaks were indexed with the Powder Diffraction Standards data (ICDD 21-1272), showing that the main structure of the samples is the anatase structure of TiO_2_. Only peaks corresponding to anatase phase were found in the XRD patterns of TiO_2_ samples.


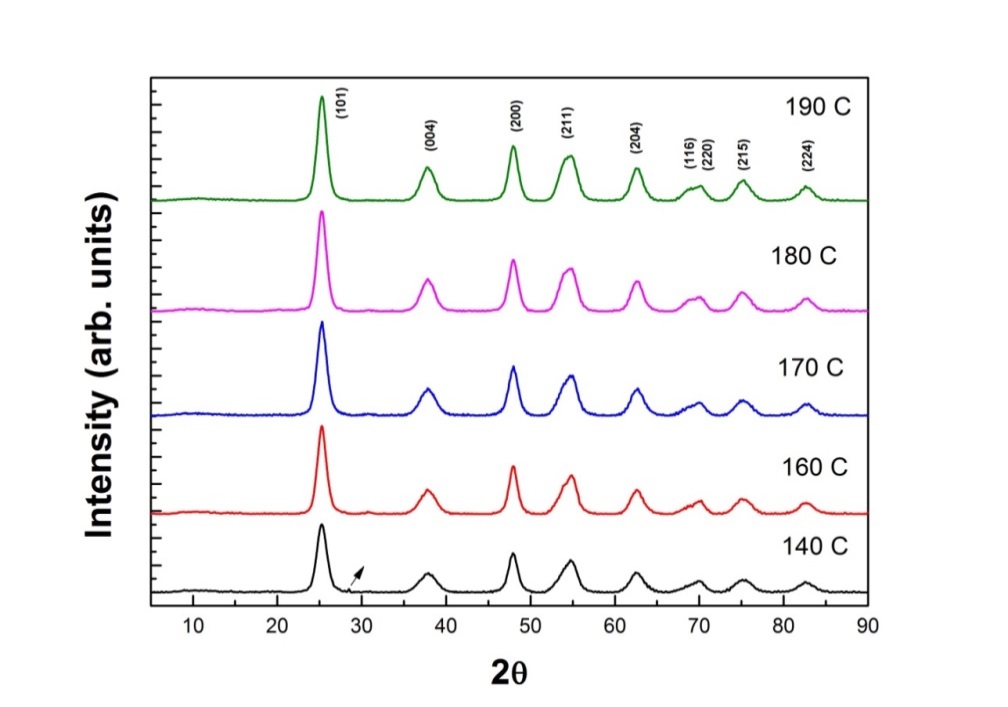


**Fig S3: XRD pattern of TiO_2_ nanoparticles prepared at different temperatures.**

The crystallite sizes of the prepared TiO_2_ nanoparticles were estimated from the broadening of the anatase (101) peak using the debye-Scherrer formula. The calculated crystallite size for TiO_2_ nanoparticles prepared at 140 °C, 160 °C, 170 °C, 180 °C and 190 °C are 6.52 nm, 7.75 nm, 6.89 nm, 7.65 nm, 7.12 nm respectively.

**FTIR spectra of the TiO_2_ nanoparticles:**

FTIR spectra of the TiO_2_ nanoparticles of coated with SA is shown in the Fig. S4. The sharp bands at 2924 and 2851 cm^-1^ are attributed to asymmetric and symmetric C–H vibrations of the methylene groups of SA and OA. The band at 1560 and 1443 cm^–1^ can be ascribed to the asymmetric and symmetric COO- stretches. The bands between 1630 cm^-1^ originate from the -OH group vibrations. The bands at 3400–3500 cm-1 correspond to the stretching vibration of hydroxyl groups, originating from the water adsorbed on the surface the nanowires.


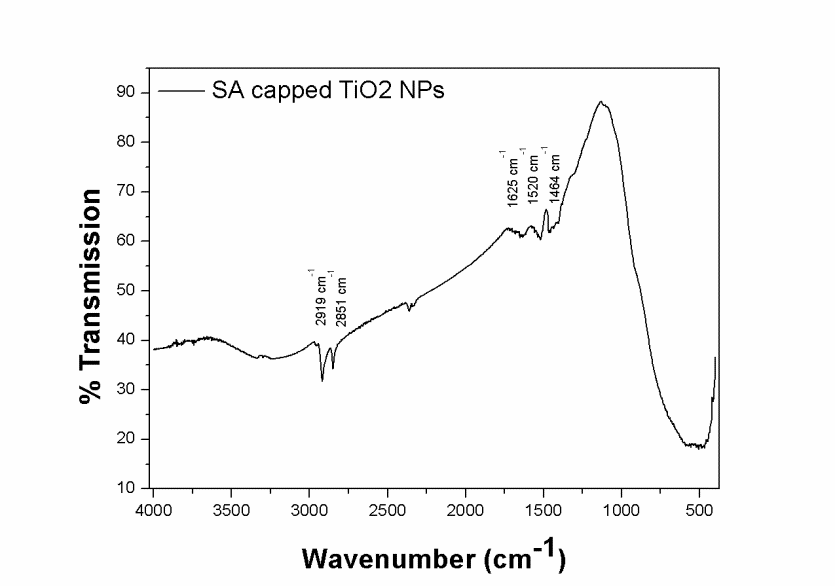


**Fig S4: FTIR spectra of the SA capped TiO_2_ nanoparticles.**
